# Including pregnant and breastfeeding people in trials of novel LAED PrEP agents: perspectives from sub‐Saharan Africa community stakeholders

**DOI:** 10.1002/jia2.26120

**Published:** 2023-07-13

**Authors:** Rhonda R. White, Molly C. Dyer, Mina C. Hosseinipour, Sinead Delany‐Moretlwe

**Affiliations:** ^1^ Science Facilitation Department FHI 360 Durham North Carolina USA; ^2^ Institute of Global Health and Infectious Disease UNC Project‐Malawi UNC School of Medicine Lilongwe Malawi; ^3^ Wits RHI University of the Witwatersrand Johannesburg South Africa

1

In 2022, the World Health Organization (WHO) recommended long‐acting injectable cabotegravir (CAB) for use as pre‐exposure prophylaxis (PrEP) [1]. This recommendation was supported by data from two large randomized controlled trials that demonstrated the safety and efficacy of CAB compared to tenofovir disoproxil fumarate and emtricitabine (TDF/FTC) for HIV prevention [2, 3]. CAB is one of the first in a pipeline of long‐acting or extended delivery (LAED) agents that have the potential to have a significant impact on high HIV incidence rates in women in sub‐Saharan Africa [4]. However, as countries plan for CAB introduction, data on the safety and pharmacology of CAB in pregnant and lactating people (PLP) are limited, presenting a challenge for programmes.

Participants of child‐bearing potential in HPTN 084, the trial that enrolled individuals assigned female at birth, were required to use long‐acting reversible contraceptives (LARCs) with a failure rate of <1%. This stringent requirement was in response to a safety signal associated with peri‐conception dolutegravir (DTG) use in women living with HIV [5]. CAB injections were discontinued in participants who declined a LARC, wished to conceive or had a positive pregnancy test, and participants were offered open‐label TDF/FTC. These actions were taken out of an abundance of caution but reinforced traditional concerns about the potential harms of including PLP in pre‐licensure trials. By contrast, there is growing support for a paradigm shift that recognizes PLP as a complex population better served through inclusion in research with adequate protection and monitoring, to generate data on safety, efficacy and pharmacology in pregnancy, thus avoiding delays in access to effective medications for PLP who also experience risk for HIV [6]. Community stakeholders, however, need to understand and support this paradigm shift before this aspiration can be fully realized.

Good participatory practice (GPP) recommends that community stakeholders contribute to protocol development to ensure locally appropriate and acceptable trial procedures [7]. Following the HPTN 084 efficacy results, all participants were unblinded, while the protocol was amended to offer access to CAB. Prior to the implementation of protocol changes, HPTN 084 community working group members hosted a stakeholder consultation to gather community perspectives on proposed pregnancy‐related amendments, including relaxation of contraceptive requirements and active CAB dosing during pregnancy and lactation. Due to COVID‐19 restrictions, a 2‐hour consultation (14:00−16:00 Central Africa Time) was held virtually. Zoom polls were used to gauge the strength of feelings around preference‐sensitive issues, to stimulate conversation and to create space to hear opposing views, and for investigators to respond to information gaps.

Overall, (*N* = 101) stakeholders from HPTN 084 countries (*n* = 82) and the United States (*n* = 19) attended the consultation, with most representing community (86%), global (7%) and other (7%) stakeholder groups as defined by GPP guidelines [7]. Despite the virtual meeting limitations, the consultation provided an opportunity to (1) provide updates on periconception DTG use and the diminishing evidence for a neural tube defect association; (2) socialize the ethical framework that reframes PLP as a complex population in need of protection through research and fair inclusion in trials; and (3) understand the acceptability of protocol amendments to community stakeholders in participant communities. An average of (*n* = 50) (range 43–52) stakeholders from sub‐Saharan Africa responded to polling questions (Figure 1). While qualitative in nature, the Zoom polls confirmed that the greatest concerns related to CAB safety during pregnancy (as opposed to lactation despite virtually no available data on CAB in breast milk), and that partners were likely to be extremely influential in participants’ decisions to use CAB in pregnancy. Subsequent discussions affirmed that the DTG experience had a negative impact on community confidence in CAB safety. This impact cannot be underestimated and will likely linger in the public consciousness. Trialists will need to provide regular communication about the evidence for risks and benefits as data evolve, but ultimately robust surveillance systems will be needed to re‐build public confidence. Despite concerns, stakeholders recognized the importance of the inclusion of PLP in research. With adequate information, stakeholders acknowledged that inclusion of PLP in trials could lead to earlier access to effective products for PLP, and avoid the harms associated with off‐label use of drugs in PLP where robust data on safety, efficacy and pharmacology are missing. While the views presented were community stakeholder views, the polls coupled with the subsequent discussions highlighted the multiplicity of strongly held opinions that a participant might have to navigate when making decisions about PrEP use in pregnancy, especially in communities where gender and social roles may limit women's autonomy and where partners place a premium on a positive pregnancy outcome. Without the consultation, it is possible that the study team could have underestimated the importance of this issue to study communities. In response, the study team developed a shareable video that showed a participant having conversations with influential members of her social network, for example partner, family members and a healthcare provider. Counselling job aids that individualize risk−benefit discussions between counsellors and participants were also developed. Tools like these may be useful in providing information to participants and their significant others about study product use in pregnancy, thus supporting choices that align with participant values while acknowledging participant autonomy and ensuring enhanced engagement in the study. While trial considerations may be unique, these tools are likely to have benefit for product introduction and programmes.

**Figure 1:**
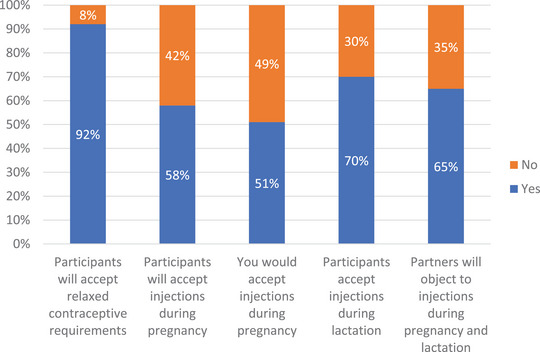
Sub‐Saharan African stakeholders’ consultation polling results.

The 2021 WHO call to action to accelerate the study of new drugs for HIV in PLP invites civil society to engage as partners in the research process, to take the lead in building community literacy and to partner with researchers to develop tools to aid communication on the need for trials in pregnancy [8]. Despite limitations, the HPTN 084 community consultation gave substance to this call and gained insights that are valuable for both trial and programme implementation. While there are unique reasons for the initial exclusion of PLP from CAB trials, the lessons from HPTN 084 are relevant for current CAB implementation and future LAED development in PLP. Safe pregnancy outcomes are a concern in many communities. Opportunities to solicit feedback, respond to concerns and build community trust in the scientific process should not be underestimated. Community stakeholders are central to efforts to include PLP in pre‐licensure trials by communicating these paradigm shifts. Over time, these engagements strengthen trial conduct, ensure support for PLP and their choices, build community confidence in LAED products and the science that confirms their effectiveness, and leading ultimately to high uptake and coverage in those at greatest need, including PLP and those that wish to conceive.

## COMPETING INTERESTS

The authors declare no competing interests.

## AUTHORS' CONTRIBUTIONS

RW, MD, SD‐M and MH designed the consultation and developed the viewpoint. SD‐M and MH also presented and addressedstakeholders' questions and comments. RW wrote first draft, and all authors contributedto revisions and approval of the final draft.

## FUNDING

This study was made possible through funding support from the National Institute of Allergy and Infectious Diseases, Office of the Director (NIAID), National Institutes of Health (NIH), Eunice Kennedy Shriver National Institute of Child Health & Human Development (NICHD), National Institute on Drug Abuse (NIDA), and the National Institute of Mental Health (NIMH), under award numbers UM1AI068619 (HPTN Leadership and Operations Center), UM1AI068617 (HPTN Statistical and Data Management Center), and UM1AI068613 (HPTN Laboratory Center). The content is solely the responsibility of the authors and does not necessarily represent the official views of the NIH. Additional funding was provided by the Bill & Melinda Gates Foundation (OPP1154174) and ViiV Healthcare. Pharmaceutical support was provided by ViiV Healthcare and Gilead Sciences.

## DISCLAIMER

The content is solely the responsibility of the authors and does not necessarily represent the official views of the National Institutes of Health.
